# Multi-element analysis of metals in human pathological and unchanged thyroid glands – pilot study

**DOI:** 10.1186/s13044-024-00197-5

**Published:** 2024-05-20

**Authors:** Aleksandra Kuzan, Justyna Rewak-Soroczyńska, Marta Kardach, Emilia Królewicz, Krzysztof Kaliszewski, Rafał Wiglusz

**Affiliations:** 1 Department of Medical Biochemistry, Wroclaw Medical University, 50-368, Wroclaw, Poland; 2 Department of Preclinical Sciences, Pharmacology and Medical Diagnostics, Faculty of Medicine, Wrocław University of Science and Technology, 50-370, Wrcław, Poland; 3 Institute of Low Temperature and Structure Research, Polish Academy of Sciences, 50-422, Wroclaw, Poland; 4 Department of General, Minimally Invasive and Endocrine Surgery, Wroclaw Medical University, Borowska Street 213, 50-556, Wroclaw, Poland; 5 Department of Organic Chemistry, Bioorganic Chemistry and Biotechnology, Faculty of Chemistry, Silesian University of Technology, Krzywoustego 4, 44100 Gliwice, Poland

**Keywords:** Thyroid, Nodular goiter, Metallomics, Glycation, Inflammation

## Abstract

Disturbances in the homeostasis of the elemental composition of thyroid tissue may have serious metabolic and health consequences. It is believed that the accumulation of some metals or the deficiency of others may even cause lethal tumours. Due to the fact that metallomics most often uses human serum to analyse macro and microelements as well as trace elements, it was decided to use material that is more difficult to obtain, but also adds credibility to the research – thyroid tissue samples biopsy. The experiments were conducted on 17 patients diagnosed with: nodular (10) and colloidal goitre (2), chronic thyroiditis (2), follicular adenoma (2) and papillary carcinoma (1). They were recruited by collecting a tumour fragment, control fragment and serum from each of them. The content of Ca, Cd, Co, Cr, Cu, Fe, Mg, Mn, Ni, Pb, Zn was examined using ICP-OES (Inductively Coupled Plasma - Optical Emission Spectrometers). Simultaneously, biochemical methods were used to determine the markers of inflammation, glycation and peroxidation: malondialdehyde, pentosidine, reactive free amine content, compounds with thiol groups and galectin 3 in the sera of the examined patients. Three statistically significant correlations were identified: Ca-Mg and Cu-Zn in control tissues (*p* < 0.05) and Cr-Mn in pathological tissues (p < 0.05). A comparison of individual groups of patients shows that there are some potentail tendencies to increase or decrease in the concentration of certain elements or markers of inflammation and glycation, therefore we discuss potential relationships between a given parameter and a thyroid disorder. The pilot study is an introduction to a deeper analysis aimed at tracing the pathomechanism of the development of thyroid diseases, so that the risk of developing these diseases can be effectively minimized.

## Introduction

The thyroid gland, which is vulnerable to the influence of environmental conditions, is an important part of the endocrine system in the human organism. Some essential chemical elements, such as: zinc (Zn), manganese (Mn), chromium (Cr), copper (Cu), iron (Fe) and lead (Pb) can be distinguished as potentially significant factors influencing the hormonal balance of the thyroid gland. The proper metabolism of thyroid hormones enables the maintenance of homeostasis. Poor physiological condition, harmful lifestyle and metal trace imbalance may predispose to the development of goiter and other thyroid diseases, including adenomas, chronic thyroiditis, and thyroid cancer [1, 2]. Goiter can be defined as a simple enlargement of the thyroid gland, it occurs in the state of iodine deficiency and hormonal imbalance. Unfortunately, the incidence of this disease is constantly increasing and nowadays it affects up to 10% people worldwide [3]. Multinodular goiter can be described as the enlargement of the thyroid probably related to environmental contaminants, genetic predispositions and other intrinsic factors [4]. Thyroid adenoma is the most common type of benign thyroid tumour, usually presents as a solitary thyroid nodule in 4% of the adult population [5, 6]. Thyroiditis is a disease characterized by a group of individual disorders causing thyroidal inflammation presenting in different ways. The most common forms of thyroiditis include Hashimoto, postpartum, and subacute [7]. Thyroid cancer is the most common solid tumour cancer which starts when cells begin to grow out of control in the thyroid gland. Thyroid cancer occurs approximately in 1.0–1.5% of all human tumours and the incidence of these carcinoma is increasing globally [8, 9]. The published studies reported that in Canada, the USA [10], Australia [11], Asia [12–15], South America [16], and Europe [17] the increase in the incidence of thyroid cancer is observed. In the past 30 years, it has been observed that papillary thyroid carcinoma represents around 90% of all diagnosed thyroid cancers [18]. In modern times, cancer and other thyroid disorders are still associated with the Chernobyl disaster that took place in 1986 [19]. Current reports (2023) indicate that it is detected the increased incidence of solid secondary tumors in men and women over a 31-year time frame after the Chernobyl disaster [20]. The consequences of this gigantic contamination of Iodine-131 (131I) mainly concerned Belarus, Ukraine, and the western part of Russia, but also concerns Poland [21].

In the well-vascularized thyroid tissue, heavy metals such as Cu, Pb, Cd tend to accumulate with a different affinity and possess a different half-life. According to the current state of knowledge, exposure to heavy metals, including cadmium and lead, may be an important factor contributing to thyroid cancer progression [22]. Moreover, it has been indicated that Pb acts as the main goitrogen, which could present its role in the unknown etiology of colloid goiter [23]. The results of research on the role of macro-, microelements and toxic metals in the development of thyroid dysfunction increase the level of knowledge about thyroid gland diseases. The observations of trace elements correlation are noteworthy in terms of autoimmunity promotion and increase of incidence of tumours. It also seems that maintaining an appropriate balance between micro- and macroelements, as well as preventing deficits of trace elements, allows the body to maintain homeostasis and contributes to increasing the effects of applied therapies [24]. It is also noteworthy that selenium and zinc are known to have anti- carcinogenic properties [25, 26]. Zinc is involved in the regulation at the T3 receptor level, while the role of copper in thyroid metabolism is not clear yet [27]. In the case of this analyte different results can be found in the literature. According to Liu serum copper concentration is positively correlated with the presence of thyroid autoantibodies [28]. Surprisingly, in another study, Cu levels have not been associated with thyroid autoimmune inflammation and thyroid autoantibodies [29].

The pilot study design included the analysis of trace elements crucial in the context of thyroid gland pathophysiology using ICP-OES (Inductively Coupled Plasma - Optical Emission Spectrometers), such as Ca, Cd, Co, Cr, Cu, Fe, Mg, Mn, Ni, Pb, Zn. The relationship between essential and/or toxic metals (Pb, Ni, Cd) as endocrine system disrupters was carefully examined.

## Materials and methods

### Biological material

The study encompassed 17 patients aged 21–68 with the diagnosis of nodular (10) and colloidal goitre (2), chronic thyroiditis (2), follicular adenoma (2), and papillary carcinoma (1). The patients were hospitalized at the Department of General, Gastroenterological and Endocrine Surgery at the Medical University of Wrocław in 2015–2018. In all cases, the diagnosis was verified as a result of a histopathological examination. The following samples were collected from each patient: an intraoperative section of the diseased thyroid gland (test sample), a section of healthy tissue (control sample), and blood serum. Blood testing routinely included measurement of thyroid-stimulating hormone (TSH), thyroxine (FT4), and glucose (data in Table 1). Patients gave their informed consent to participate in the study. The study was approved by Bioethics Committee at Wroclaw Medical University (No. 419/2022). All the measures were performed in agreement with the ethical standards of the Helsinki Declaration.

**Table 1:** Basic information about the examined group

	Mean	Median	Minimum	Maximum	Lower Quartile	Upper Quartile	Standard deviation
age [years]	50.82	54.00	21.00	68.00	39.00	64.00	15.46
TSH [uIU/ml]	1.70	1.30	0.65	7.73	0.88	1.57	1.72
FT4 [pmol/l]	14.44	14.75	11.10	16.70	12.90	16.10	1.88
creatinine [mg / dl]	0.74	0.72	0.54	1.35	0.63	0.80	0.18
glucose [mg / dl]	98.81	92.50	80.00	140.00	90.00	102.75	18.33

### ICP-OES coupled plasma optical emission spectrometer

The intraoperative sections of the thyroid glands weighing approx. 32.5 mg (+/− 13.06 mg) were subjected to the procedure described in Kuzan et al. 2021 [30]. The samples were transferred to a Teflon vessel containing 1 M ultrapure HNO_3_ (Sigma-Aldrich, Saint Louis, MI, USA) and placed in a microwave reactor for 90 min. In microwave-stimulated hydrothermal conditions, under autogenous pressure of 25 atm and at 250 °C, the procedure was repeated for every specimen. The applied ICP-OES measurement procedure is detailed in Kuzan et al. 2021b [30]. In brief, the sample solutions prepared by the wet digestion in the closed-vessel microwave-assisted system were analyzed using an Agilent benchtop synchronous vertical dual view (SVDV) ICP-OES instrument, model 5110. To introduce the solutions (samples and standards), the instrument was equipped with an easy-fit quartz torch with a standard 1.8 mm injector and a Seaspray nebulizer mounted into a double-pass glass cyclonic spray chamber. Six simple (with no matrix matching) multielement standard solutions were applied for the calibration of the instrument, the concentrations of these standards was as follows: 0.10, 0.50, 1.00, 2.00, 5.00 and 10.00 μg/ml. The determination coefficients for these calibration curves were in the range 0.991 to 1.000, pointing the linearity. The instrument was run under the operating conditions recommended by the manufacturer. These working parameters are given in Table 2. All samples solutions were measured 3 times, hence, the results used for the statistical evaluation were the average values. The relative standard deviations acquired for these measurements was fairly good and changed from < 0.5 to 10%, depending on the concentration of the studied elements. The accuracy was controlled by incorporating into the measurement sequences 3 quality control (QC) samples, being the standard solutions containing verying concentrations of the analytes, i.e., low (0.20 μg/ml), medium (2.50 μg/ml) and high (8.0 μg/ml). The recoveries assessed for the studied elements, when measuring these QC samples, was in the range from 91 to 106%, hence, was also acceptable. When these recoveries were lower than below 90%, the instrument was recalibrated.”

**Table 2:** The working parameters for the ICP-OES instrument used in this work (Agilent 5110)

RF power (kW): 1.2	
Plasma flow rate (L min^−1^): 12.0	
Auxiliary flow rate (L min^−1^): 1.00	
Nebulizer flow rate (L min^−1^): 0.70	
Standard pump speed (rpm): 12	
Fast pump speed (rpm): 50 (during solution uptake and rinsing)	
Viewing mode: axial	
Replicate read time (s): 3	
Stabilization time (s): 15	
Sample uptake delay time (s): 10	
Number of replicates: 3	
Background correction: fitted (to correct for the spectral interferences)	
Analytical lines (nm): Ca II 396.85, Cd II 214.4, Co II 238.9, Cr II 267.7, Cu I 327.40, Fe II 238.20, Mg II 279.55, Mn II 257.61, Ni II 231.6, Pb 220.4, Zn I 213.86	

I and II denote the atomic and ionic lines, respectively

In the case of the analytes for which the result below the detection of the applied ICP-OES method was obtained, the LOD (limit of detection) was determined. In this case, the 3σ criterion was used, where σ is the standard deviation for 10 subsequent measurements of the procedural blanks resulting from the wet digestion sample preparation step. These 3σ values were then expressed as the concentrations, using the slopes of the calibration curves acquired, and are presented in Table 3 for Cd, Co, Cr, Mn, Ni and Pb (the elements which concentrations were very low and in many cases undetected). In addition, the LOD values for other elements studied (Ca, Cu, Fe, Mg, and Zn) are included.

**Table 3:** The LOD values (in ng/ml) for Ca, Cd, Co, Cr, Cu, Fe, Mg, Mn, Ni, Pb and Zn

Cd	Co	Cr	Mn	Ni	Pb
0.40	3.5	0.55	0.20	34	6.0
Ca	Cu	Fe	Mg	Zn	
45	0.70	5.0	1.6	3.0	

Results were converted from μg / ml to μg / mg of tissue.

### Assessment of the content of compounds with thiol groups (glutathione)

As described in Kuzan et al. 2022 [31], the reaction between compounds containing thiol groups from patients’ serum and 0.1 M 5,5-DiThio-bis-2-NitroBenzoic acid (DTNB) in 10 mM sodium-phosphate buffer pH 8.00 was performed in the presence of 10% Sodium Dodecyl Sulfate (SDS) for 60 minutes at 37 °C. Absorbance against a blank sample at λ = 412 nm was measured. On the basis of the data from the standard curve, the content of the thiol group was calculated in the examined material.

### Analysis of GAL3 protein content

The concentration of GAL3 in serum samples was performed using human immunoassay ELISA kits from Elabscience® (Elabscience Biotechnology Inc., Houston, Texas, United States). The manufacturer’s instructions were followed.

### Assessment of lipid peroxidation based on the determination of malondialdehyde (MDA) in serum

As described in Kuzan et al. 2022 [31]. The mixtures consisting of serum, 15% trichloroacetic acid (TCA) solution in 0.25 M hydrochloric acid (HCl) and 0.37% thiobarbituric *acid (*TBA) in 0.25 M HCl were incubated for 20 minutes at 100 °C, then cooled down, rotated for 5 minutes at 4700 RPM. The absorbance of supernatant was measured at 535 nm. The calculation of MDA concentration was performed using the Lambert-Beer law, at absorbance coefficient ε = 156 mmol^−1^· L · cm^−1^.

### Reactive free amine content (RFA)

As described in Kuzan et al. 2022 [31], serum samples were diluted 200-fold with 50 mM carbonate buffer (pH 10.5). Then, 9-μL aliquots of the diluted specimens were pipetted into a 96-well microplate and 91 μL of freshly made o-phthalaldehyde (OPA) solution (5 mg OPA, 100 μL pure ethanol, 5 μL β-2-mercaptoethanol, following this 10 mL 50 mM carbonate buffer pH 10.5) was added to all wells. The samples were read immediately at 340 nm excitation and 455 nm emission wavelengths.

### Pentosidine

As described in Kuzan et al. 2022 [31], the serum samples were diluted 100-fold with 0.9% natrium chloratum (NaCl). The absorbances of these samples at λ = 280 nm were measured. To assess the total pentosidine content in the sample, the fluorescence of the samples was measured at excitation wavelength 335 nm and emission 385 nm on EnSpire Multimode Plate Reader (Perkin Elmer, Waltham, MA, USA). Fluorescence was divided by Abs 280 assuming the resulting values as data expressed in arbitrary units.

### Statistics

The statistical analysis was conducted using the data analysis software system Statistica 13.3 (TIBCO Software Inc., Palo Alto, California, USA) software. The statistical significance level was set at *p* < 0.05. The Spearman correlation test, the Wilcoxon pairwise test, the Kruskall-Wallis ANOVA test, and the Mann-Whitney test were used. In the case of questions requiring multiple comparisons, the Bonferroni correction was applied.

## Results

The analysis showed that there was no detectable amount of cadmium, cobalt, nickel or lead in any of the samples in investigated biological material. Chromium and manganese were detectable only in some samples (chromium in 22/34 tissue sections, manganese in 26/34 tissue sections). The other analysed analytes - the content of calcium, copper, iron, magnesium, and zinc in relatively high concentrations were observed in each sample. The table collecting the basic descriptive statistics is attached as Table 4.

**Table 4:** Summary of metal concentrations in the sample of thyroid tumours (−t), controls (−c), and other analytes in the serum

	N	Mean	Median	Lower Quartile	Upper Quartile	Standard deviation
Ca-t [ug/mg]	17	0.2188	0.2044	0.0767	0.4135	0.1715
Cu-t [ug/mg]	17	0.0056	0.0044	0.0018	0.0133	0.0038
Fe-t [ug/mg]	17	0.1220	0.1094	0.0585	0.2432	0.0884
Mg-t [ug/mg]	17	0.0261	0.0235	0.0127	0.0484	0.0200
Zn-t [ug/mg]	17	0.0192	0.0167	0.0096	0.0424	0.0142
Ca-c [ug/mg]	17	0.2336	0.1939	0.1020	0.6560	0.1439
Cu-c [ug/mg]	17	0.0063	0.0046	0.0030	0.0157	0.0039
Fe-c [ug/mg]	17	0.0244	0.0209	0.0121	0.0595	0.0163
Mg-c [ug/mg]	17	0.0218	0.0200	0.0118	0.0431	0.0151
Zn-c [ug/mg]	17	0.1318	0.1234	0.0941	0.2436	0.1082
thiol groups [nM]	17	78.6529	50.1000	0.0000	434.2000	28.1000
GAL-3 [ng/ml]	17	12.1853	12.2800	1.1400	18.2500	10.5700
MDA [nM]	17	0.5285	0.5400	0.1690	0.8100	0.4080
RFA [AU]	17	62,177.9482	63,571.8800	34,048.8800	77,789.3800	53,520.3800
pentosidine [AU]	17	1067.1941	923.6000	164.4000	2139.2000	814.5000

After applying the Bonferroni correction, significant correlations can be observed for the following pairs of variables (Table 5).

**Table 5:** Correlations that are statistically significant or close to significance between the analysed parameters

	N (Significant)	R (Spearman)	t(N-2)	p	significance after Bonferroni correction
Cr-t [ug/mg] & Mn-t [ug/mg]	17	0.723173	4.05526	0.001036	Yes
Ca-c [ug/mg] & Mg-k [ug/mg]	17	0.781863	4.85707	0.001461	Yes
Cu-c [ug/mg] & Zn-c [ug/mg]	17	0.708333	3.88645	0.001036	No
Ca-t [ug/mg] & pentosidine [AU]	17	−0.536765	−2.46391	0.026312	No
Ca-c [ug/mg] & pentosidine [AU]	17	−0.563725	−2.64334	0.018435	No
Mg-t [ug/mg] & pentosidine [AU]	17	−0.639706	−3.22340	0.005685	No
Mg-c [ug/mg] & pentosidine [AU]	17	−0.571078	−2.69435	0.016646	No
Mn-t [ug/mg] & RFA [AU]	17	0.545017	2.51762	0.023667	No
Cu-t [ug/mg] & GAL-3 [ng/ml]	17	0.578431	2.74632	0.014997	No

**Fig. 1:**
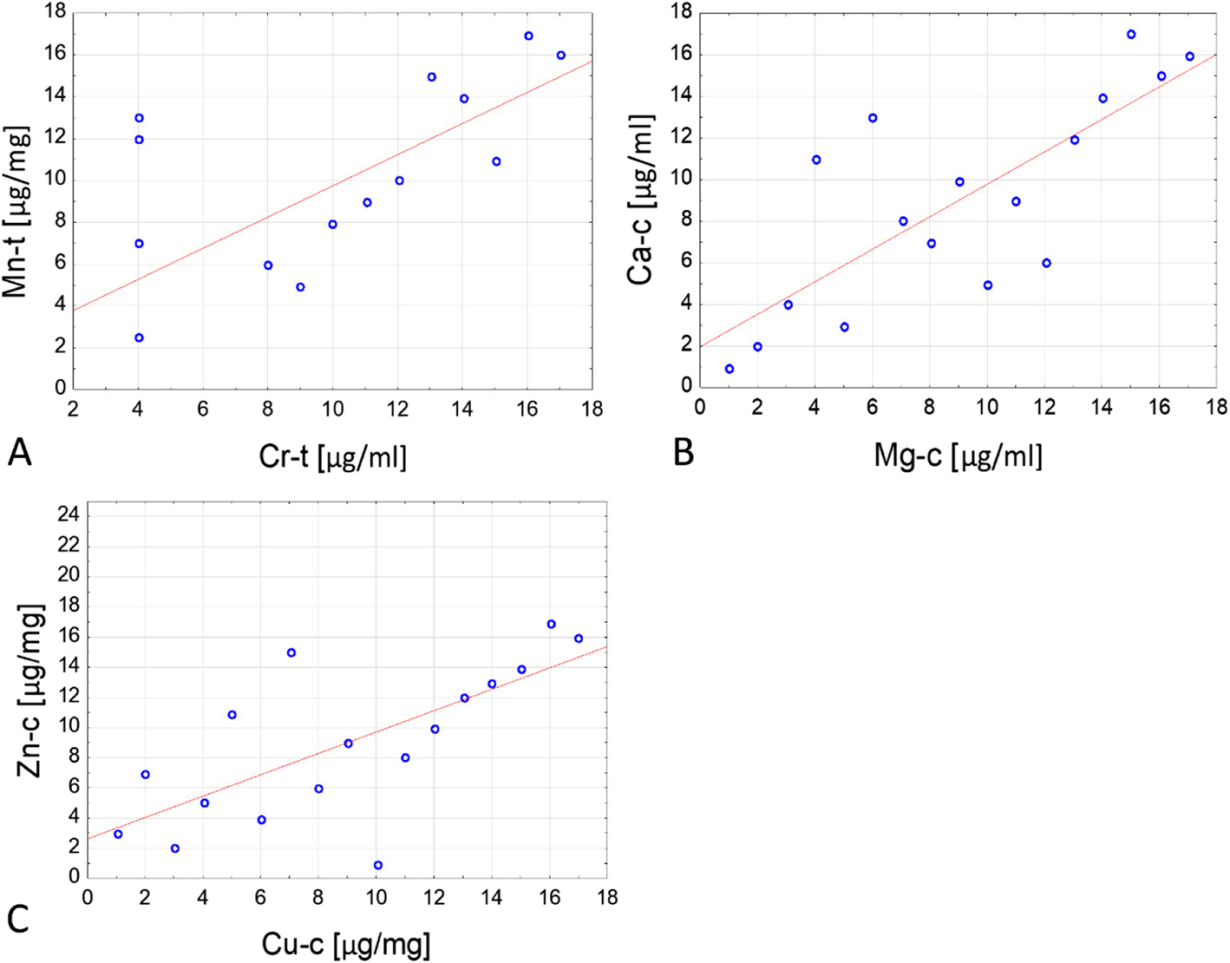
Graphs of correlation for statistically significant analytes

Correlation plots were prepared for these pairs of variables based on the ranks of observations (Fig. 1).

**Fig. 2:**
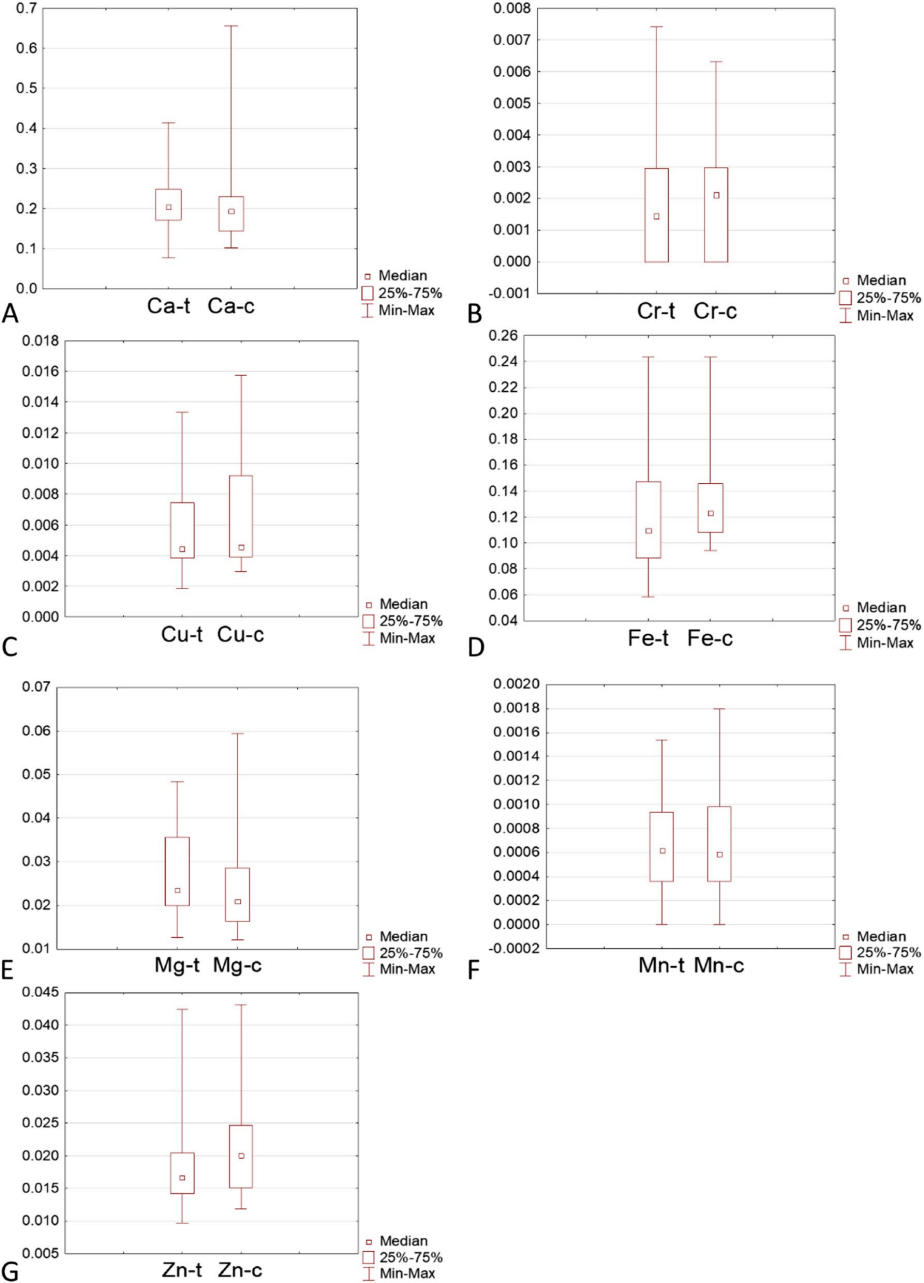
Comparison of control samples and tumours in terms of the content of selected metals in the tissue

Based on the comparison of the elemental composition of the tissue sections of pathologically changed and control tissues, it was found that there is no possibility to confirm the research hypothesis. In the pathologically altered thyroid there is an accumulation of heavy metals and/or a decrease in the concentration of metals supporting the antioxidant properties of molecules. A graphical representation of these data is shown in Fig. 2, it can be seen that the medians for the control and for tumours have similar values and do not differ statistically from each other.

**Fig. 3:**
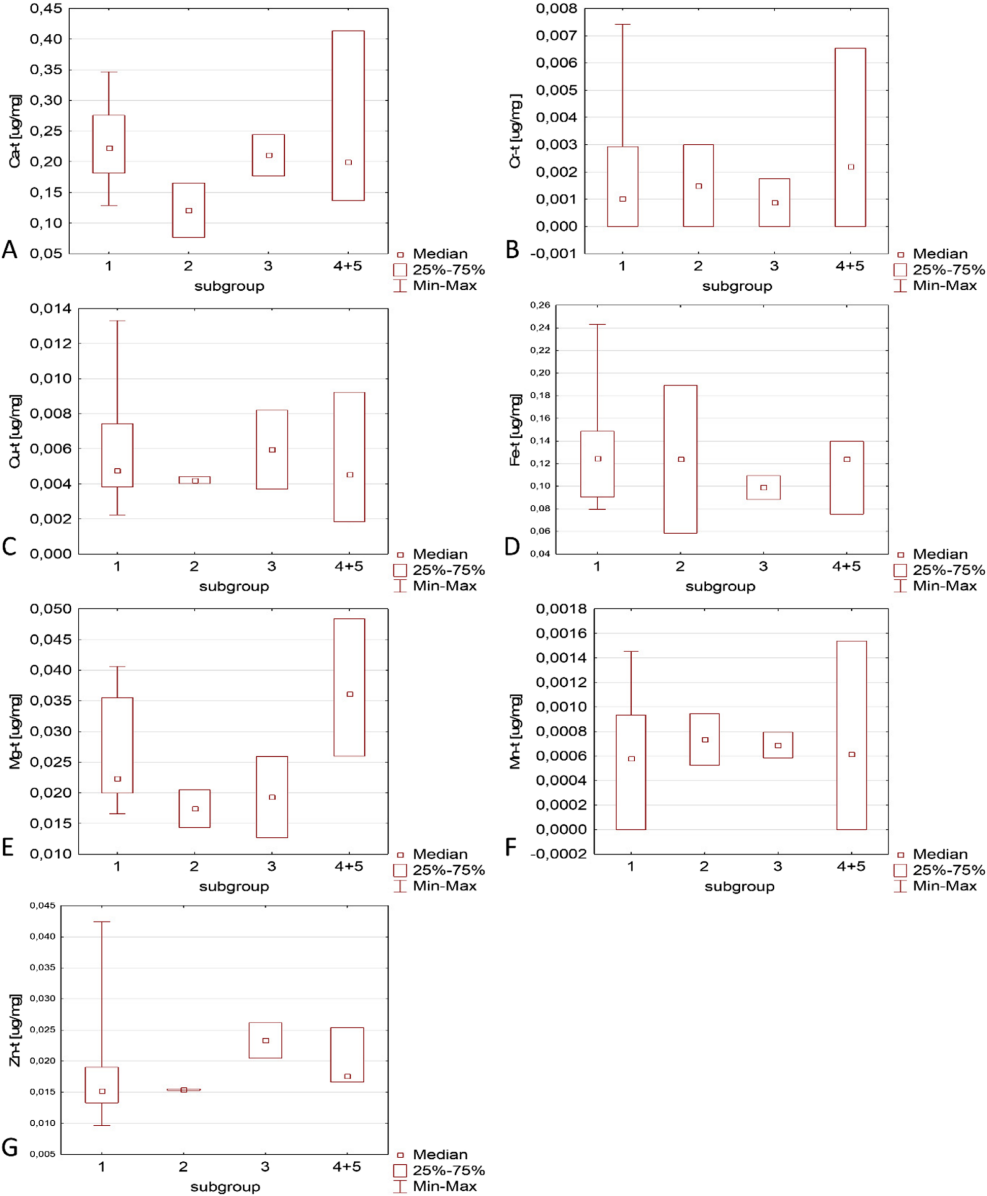
Comparison of various types of tumours in terms of the content of selected metals in the thyroid tissue. The subgroups are marked as: 1- nodular goitre, 2- thyroiditis, 3- colloid goiter, 4 and 5- follicular and papillary adenoma (combined group)

**Fig. 4:**
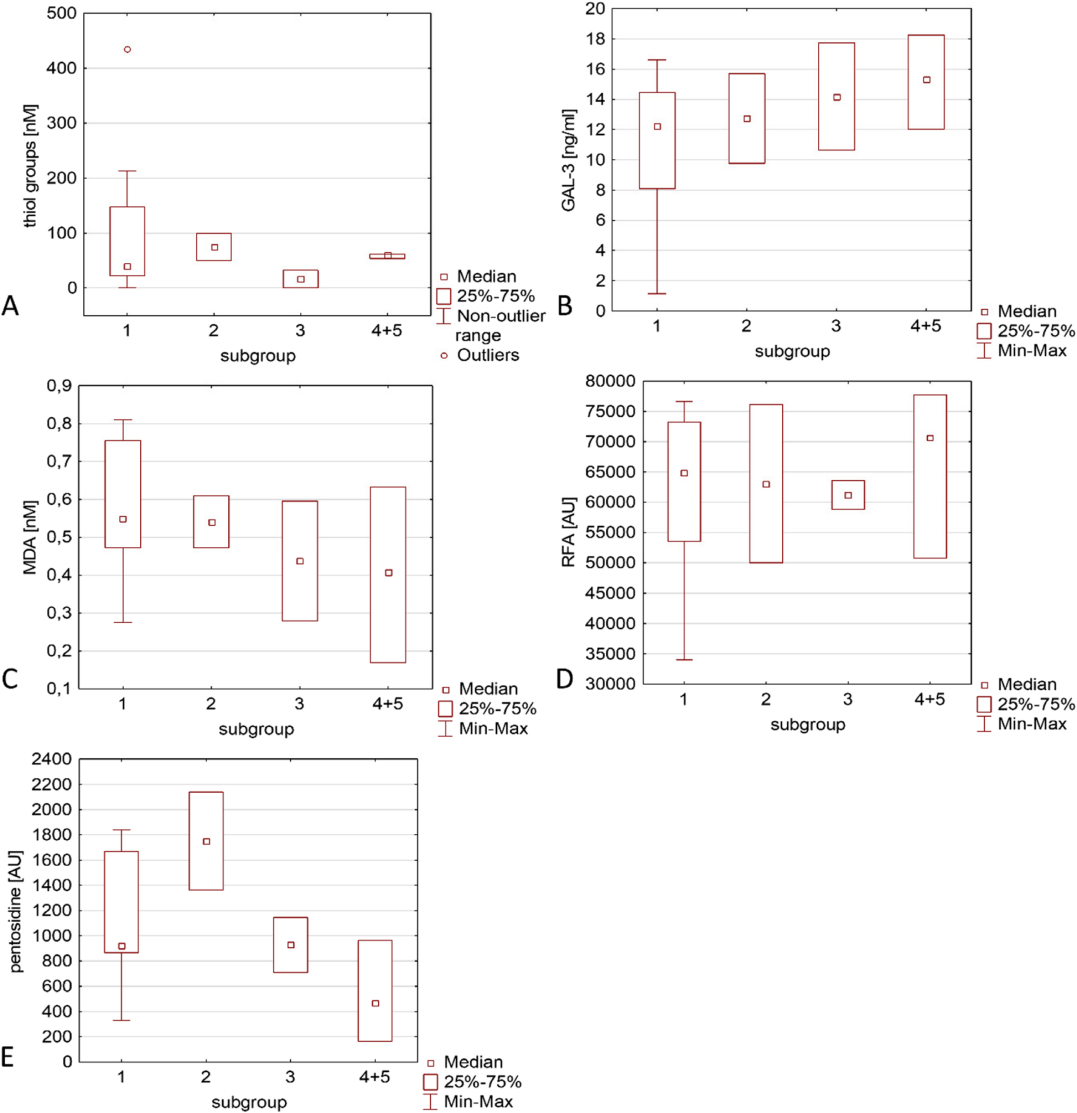
Comparison of various types of tumours in terms of the content of various serum biochemical parameters of patients with thyroid pathologies. The subgroups are marked as: 1- nodular goitre, 2- thyroiditis, 3- colloid goiter, 4 and 5- follicular and papillary adenoma (combined group)

The comparison of the different types of thyroid pathology in terms of the content of different metals and the biochemical parameters of patients’ blood is shown in Figs. 3 and 4. The small number of samples in most groups did not allow for the identification of statistically significant relationships. We can only demonstrate some trends, for example, that pentosidine seems to be a parameter more related to inflammation of the thyroid gland than thiol groups or lipid peroxidation. In contrast, chromium, calcium, and zinc appear to be less abundant in typical inflammatory tissue samples compared to other types of pathological tissues. Magnesium appears to occur in larger quantities in adenomas than in other types of tumours. There seems to be a relatively high amount of zinc in the goiter colloid samples, compared to other tissues. Since this is a pilot study, it is obvious that it should be repeated on a much larger number of samples in order to finally reveal dependencies or conclude that they are absent.

## Discussion

Typically, the contents of the chemical elements in relation to thyroid pathologies can be detected in serum, blood, plasma or urine. In this century, similar research was conducted by Boulyga et al. (2000) and Stojsavljević et al. (2020). The former had samples from patients living in the Chernobyl area [32], so it is hard to expect here that the results will be congruent. Stojsavljević et al. wrote three publications, one of which deals with the elemental composition of blood [1], while the remaining/other two focused on tissue analysis. Stojsavljević et al. compared control thyroid glands only to Hashimoto’s thyroiditis sections [33]. Unfortunately, the panel of elements that they examined did not fully coincide with the one we analysed, for example, there is no chromium or calcium in it. However the analysis of the correlation between analytes from thyroid tissue coincides with our observations regarding the Cu-Zn (at the level of r approx. 0.7) [33].

The second similar work by Stojsavljević et al. concerned the comparison of neoplastic, colloidal and control samples. Comparing to control samples they found in the goiter a reduced content of basic elements (Mn and Se) and an increased content of toxic metals (Pb, Th and U) [23]. In our examination Mn did not turn out to be lower in goiter than the rest of the samples, however, we did not analyse Se. The exposure of the patients to lead was lower, because in our examined group the presence of this element in the thyroid was not observed at all. It can be seen that the differences in conclusions may be largely related to the group of patients to be recruited – their the surrounding industry, water contamination and air pollution, the waste policy of a given country and probably the eating habits of a given group of patients, and many others factors.

Another study that analysed the content of cadmium in human thyroid tissue concerned the examination of autopsy samples, it was published in 1995 and showed that there was a relationship between age and the content of this metal in the thyroid gland, without analysing the state of the thyroid gland, which made the conclusion questionable [34]. No relationship with age was found in our study.

Our results show that there are no statistical differences between the levels of metals in the tissues of neoplastic tumours and benign thyroid tumours or control thyroid sections are inconsistent with the hypothesis, but consistent with the results of some other authors. According to the meta-analysis by van Gerwen et al. there was a significant difference in medians only in Mn tissue levels between thyroid cancer patients and benign thyroid patients. For the other included tissue, metal ions compared between thyroid cancer and benign thyroid patients, and also between thyroid cancer patients and healthy controls, no significant medians differences were found, including the known carcinogenic metal ions included in the meta-analysis (As, Ni and Cr) [35].

Among the correlations identified in this study, the Cu-Zn relationship seems to be particularly important. As mentioned above, the same relationship was found in another similar study on the thyroid glands [33], but also in other tissues, such as arteries [30]. Both of these metals play a key role in metabolism related to protection against oxidative stress. Together they form a component of the active enzyme superoxide dismutase (Cu, Zn-SOD) neutralizing reactive oxygen species. In addition, zinc has a large effect on the functioning of the thyroid gland due to the fact that it is required for the synthesis of the thyrotropin-releasing hormone (TRH), and for the conversion of thyroxine (T4) to triiodothyronine (T3), it plays an important role in the binding of T3 to its nuclear receptor and participates in the synthesis of the thyroid-stimulating hormone (TSH) [2, 36]. Copper has a similar effect –it stimulates the production of T4 and prevents over absorption of T4 by controlling the calcium levels [37]. The fact that Cu influences the pathology of the thyroid gland is also evidenced by the described here correlation between Cu and Gal3 –a recognized marker of thyroid cancer [38] and other pathological conditions such as heart failure [39]. No wonder then that disturbances in the content of copper and zinc will result in malfunctions in the work of the thyroid gland. In their meta-analysis, Gumulec et al. found significantly decreased zinc levels in the thyroid tissue of patients with thyroid cancer compared to control patients [40]. In this study, there is a tendency for a decrease in zinc and copper in the inflamed gland, but an increase in Zn in the case of colloidal goiter. Therefore, it can be very cautiously concluded that it is necessary to balance the content of these elements, using the possible supplementation of Zn, but avoiding overdosing.

The calcium-magnesium correlation is also reported in other tissues, including arteries [30]. We suspect that this relationship is simply due to the chemical similarity of these two elements: both exist as bi-positive ions, both have structural functions, apart from regulatory functions, and both are part of various salts that stiffen the tissue. It is reported that magnesium may mimic calcium ion activity [41]. The imbalance in the content of these elements has systemic effects, however, it is strongly related to the thyroid gland: hypercalcemia is a condition that accompanies cancer or hyperthyroidism, and hypocalcemia can develop after the surgical removal of the thyroid gland with damage to the parathyroid glands [41]. It is postulated that Mg deficiency might be associated with the inflammation or increased levels of free radicals, which might lead to oxidative DNA damage and cancer formation, because magnesium stabilizes the structure of nucleic acids and is a vital cofactor of enzymes involved in DNA replication, repair, and gene expression [37]. In our study, we see a tendency for adenomas to contain less magnesium, and less calcium in typical inflamed tissues.

The next correlation that emerged concerns chromium and manganese. Please note that it applies only to tumours - no statistically significant relationship was found in the control samples. The more chromium, the more manganese is also found in the human serum of people with thyroid diseases, unlike the serum of healthy people, where such a relationship is not observed [42]. Cr and Mn have many common features – they are transition metals, occurring in many degrees of oxidation (chromium: + II, + III and + VI, manganese: + II, + IV and + VII). Both chromium and manganese compounds (with a higher oxidation state) are strong oxidants, participate in various types of oxyreduction reactions catalysed by enzymes, for example, Mn-SOD. An excess of manganese can even be toxic to the thyroid gland [43]. This is also evidenced by the result of this project, where the correlation close to the significance Mn-RFA was obtained. It can therefore be concluded that the more manganese in the tissue, the greater the oxidative stress.

Returning to the Mn-Cr correlation in tumours –the elements are known to be an essential trace nutrient, however, they turn out to be potentially toxic at high levels of exposure. So far no answer has been found to the question about the molecular mechanism responsible for the occurrence of this correlation, which means that it is a promising research direction leading to a deeper understanding of pathomechanims leading to the thyroid disease.

An interesting result of this project was the discovery of a negative correlation close to significance between Mg and Ca and pentosidine. The question arises whether Mg and Ca protect against glycation. There are studies that prove that Mg and Ca supplementation has a positive effect on sugar metabolism in patients with type 2 diabetes, as it influences β-cell functioning and acts as a cofactor of many enzymes involved in the glucose level lowering [44]. Intensive glycation, leading to the formation of advanced glycation end products (AGEs), including pentosidine, is a very unfavourable process, which can also initiate and accelerate cancer formation, including the thyroid gland cancer [45, 46]. If the anti-glycation effects of calcium and magnesium are confirmed, it will be important information for endocrinologists dealing with diabetes and thyroid diseases.

The biggest limitation of the study is the small number of samples. We emphasize, however, that this work was to be a pilot study to prepare the methodology for a larger study and to initially check whether the trends described by the authors of other publications will be visible in our small population. The great advantage of this study are, however, the parallel biochemical tests that we compared and analysed with the level of various metals. It turned out that in such a small group we cannot confirm the hypothesis that in neoplastic and inflammed tissues the level of glycation, peroxide and inflammation in general is much higher, but we have outlined a direction that seems worth following.

## Data Availability

The datasets used and analysed during the current study available from the corresponding author on reasonable request.
